# Long-Term Outcomes of Mucosal Early Gastric Cancer with Lymphatic Invasion as the Sole Non-Curative Factor After Endoscopic Submucosal Dissection

**DOI:** 10.3390/cancers18101653

**Published:** 2026-05-20

**Authors:** Na-Kyung Lee, Tae-Se Kim, Soomin Ahn, Yang Won Min, Hyuk Lee, Byung-Hoon Min, Jun Haeng Lee, Poong-Lyul Rhee

**Affiliations:** 1 Department of Medicine, Samsung Medical Center, School of Medicine, Sungkyunkwan University, 81 Irwon-ro, Gangnam-gu, Seoul 06351, Republic of Korea; 2nk627@gmail.com (N.-K.L.);; 2 Department of Pathology and Translational Genomics, Samsung Medical Center, School of Medicine, Sungkyunkwan University, 81 Irwon-ro, Gangnam-gu, Seoul 06351, Republic of Korea

**Keywords:** stomach neoplasms, early gastric cancer, endoscopic submucosal dissection, lymphatic metastasis, lymphatic invasion

## Abstract

Background: The clinical significance of lymphatic invasion in mucosal early gastric cancer (EGC) treated with endoscopic submucosal dissection (ESD) remains unclear. We evaluated clinicopathologic features and long-term outcomes in patients with lymphatic invasion as the sole non-curative factor. Methods: We retrospectively reviewed 9117 patients who underwent ESD for EGC at Samsung Medical Center between 2001 and 2022. Among patients with mucosal disease and lymphatic invasion as the sole non-curative factor, long-term clinical outcomes were summarized using an outcome flowchart, and characteristics of lymph node-positive cases were analyzed in relation to curative resection criteria. Results: Among 7444 patients with mucosal EGC treated with ESD, lymphatic invasion was identified in 154 patients (2.1%). Among the 117 patients with lymphatic invasion as the sole non-curative factor, the overall rate of pathologically confirmed or clinically suspected lymph node metastasis (LNM) was 4.3% (5/117). Specifically, LNM was identified in 3.2% (3/95) of patients who underwent additional surgery, and in 9.0% (2/22, including one clinically suspected case) managed with observation alone during a median follow-up of 58.0 months. LNM was observed exclusively in lesions involving the muscularis mucosae or in lesions larger than 2 cm, whereas no LNM occurred in tumors confined to the lamina propria measuring ≤ 2 cm. Conclusions: Despite mucosal confinement, lymphatic invasion was associated with a clinically meaningful risk of LNM, whereas no LNM was observed in lesions ≤ 2 cm confined to the lamina propria. For patients with mucosal EGC in whom lymphatic invasion is the sole non-curative factor, careful, individualized decision-making is warranted.

## Simple Summary

The clinical significance of lymphatic invasion (LI) as a solitary non-curative factor in mucosal early gastric cancer (EGC) after endoscopic submucosal dissection (ESD) remains unclear. In this study of 9117 patients, we found that although LI is rare (2.1%), it carries a meaningful risk of pathologically confirmed or clinically suspected lymph node metastasis (LNM) (4.3%), even when the tumor is confined to the mucosal layer. Notably, LNM was observed only in tumors involving the muscularis mucosa or larger than 2 cm, whereas no LNM occurred in tumors ≤ 2 cm confined to the lamina propria. Our findings suggest that while additional surgery is generally recommended for LI-positive cases, a more tailored approach may be appropriate for specific low-risk patients.

## 1. Introduction

Endoscopic submucosal dissection (ESD) is an established standard treatment for early gastric cancer (EGC) when the risk of lymph node metastasis (LNM) is negligible. However, when non-curative resection occurs after ESD, additional gastrectomy with lymph node (LN) dissection is generally recommended because of the clinically meaningful risk of LNM and poorer prognosis [1,2,3,4,5]. Therefore, identifying patients at high risk of LNM is crucial for determining the necessity of additional surgery [6,7].

Lymphovascular invasion (LVI) is a well-established risk factor for LNM and unfavorable survival outcomes. Accordingly, LVI is classified as a non-curative factor after ESD and is assigned the highest risk score (3 points) in the eCura system [8,9]. Previous studies have reported significantly lower 5-year survival rates in LVI-positive patients [10,11]. Moreover, recent data indicate that extra-gastric metastasis (EGM) occurs in approximately 6.4% of LVI-positive patients despite R0 resection, suggesting that the risk of recurrence cannot be completely excluded [12].

In contrast, our group previously suggested that additional surgery after endoscopic resection may be unnecessary in patients with LVI who meet the absolute criteria for endoscopic resection [13]. Additionally, Matsueda et al. reported LNM in only 1 of 40 patients (2.5%) with pT1a gastric cancer and lymphatic invasion (LI) after ESD, particularly among differentiated-type lesions [14]. However, most previous studies evaluating LVI included heterogeneous cohorts combining mucosal and submucosal EGC or analyzed LI in conjunction with other non-curative factors [6,8,9,15,16,17,18,19,20].

Taken together, despite accumulating evidence on LI after ESD, the independent clinical significance of LI in mucosal EGC, particularly when it is the sole non-curative factor, remains insufficiently defined. This study aimed to investigate the clinicopathologic features and long-term clinical outcomes of mucosal EGC with LI as the sole non-curative factor after ESD.

## 2. Materials and Methods

### 2.1. Study Design and Patients

**Figure 1:**
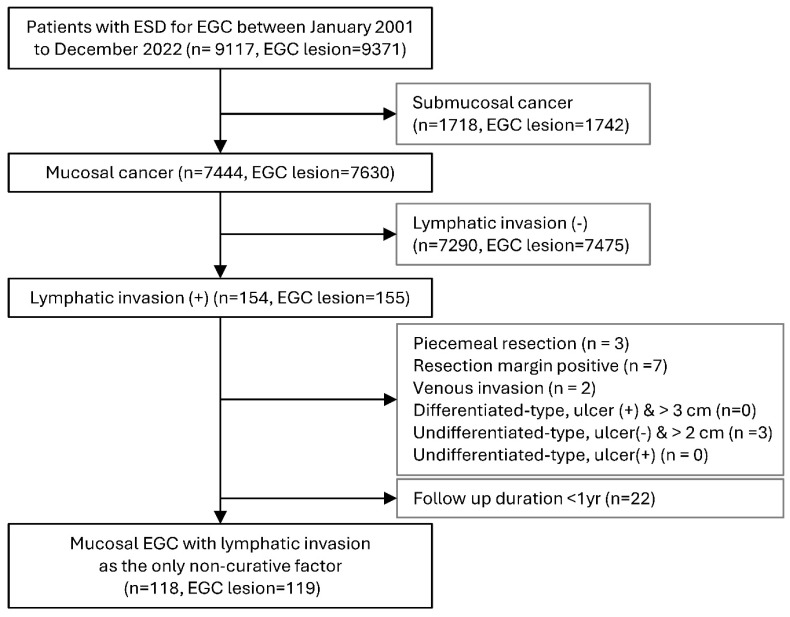
Patient flowchart. ESD, endoscopic submucosal dissection; EGC, early gastric cancer; yr, year.

**Figure 2:**
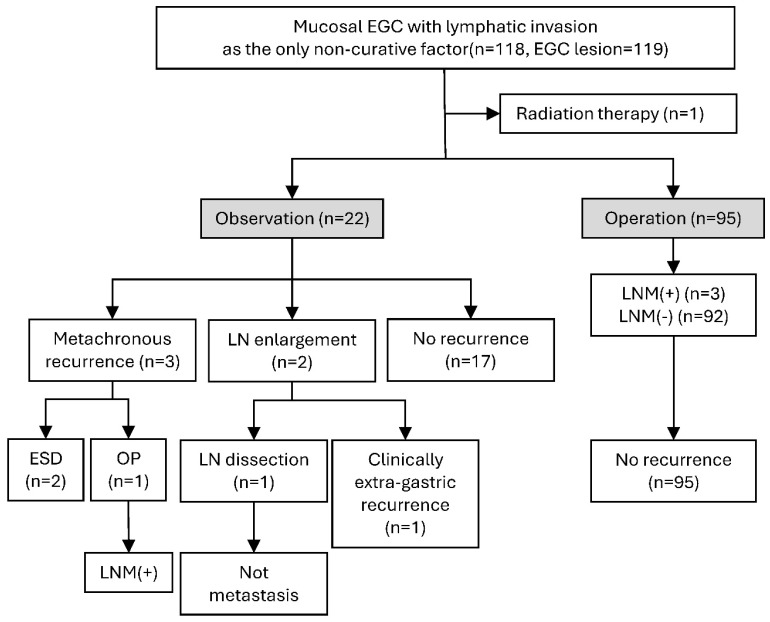
Outcomes of cases with lymphatic invasion as the sole non-curative factor. EGC, early gastric cancer; LNM, lymph node metastasis; LN, lymph node; ESD, endoscopic submucosal dissection; OP, operation.

This was a retrospective cohort study conducted at Samsung Medical Center. From January 2001 to December 2022, a total of 9117 patients (9371 lesions) underwent ESD for EGC. Among them, 1718 patients (1742 lesions) with submucosal cancer were excluded. Of the remaining 7444 patients (7630 lesions) with mucosal EGC, 154 patients (155 lesions) with LI were compared with 7290 patients (7475 lesions) without LI to identify clinicopathologic characteristics associated with LI. To evaluate the long-term outcomes of mucosal EGC with LI as the sole non-curative factor, only patients with mucosal EGC and LI as the sole non-curative factor were included. Patients with additional non-curative factors (*n* = 15) or a follow-up duration of less than 1 year (*n* = 22) were excluded. Consequently, 118 patients with 119 mucosal EGCs with LI as the sole non-curative factor were included in the study cohort (Figure 1). After excluding one patient who received radiotherapy (RT), the final analytic cohort consisted of 117 patients (118 lesions) (Figure 2).

The study was approved by the Institutional Review Board of Samsung Medical Center, Seoul, Korea (approval no. 2026-01-102). The requirement for written informed consent was waived due to retrospective analysis of de-identified data. The study adhered to the principles of the Declaration of Helsinki.

### 2.2. ESD Procedure and Pathologic Evaluation

The ESD procedures conducted at our institution have been previously described [21,22]. All ESD procedures were performed by board-certified gastroenterologists experienced in gastric ESD at Samsung Medical Center. ESD procedures were performed using standard single-channel endoscopes, including GIF-Q260J, GIF-Q260, GIF-240EGD, and GIF-Q240 endoscopes (Olympus Medical Systems, Tokyo, Japan). All resected specimens were stretched, secured to a polystyrene plate, and fully immersed in 10% neutral buffered formalin for at least 12 h to ensure adequate fixation. After fixation, the specimens were serially sectioned at 2 mm intervals, parallel to the closest resection margin to assess both lateral and vertical margins as recommended by the Korean pathology guidelines [23]. Histopathologic evaluation included assessment of the lateral and vertical resection margins, depth of tumor invasion, presence of ulceration, and LVI. All pathological evaluations were performed by experienced gastrointestinal pathologists. LI was first determined using hematoxylin and eosin (H&E)-stained sections. When the presence of LI was equivocal on routine histologic examination, additional immunohistochemical staining with a D2-40 monoclonal antibody was performed to confirm LI [24].

### 2.3. Definition

The curability of ESD in mucosal cancer was based on the Korean practice guidelines for gastric cancer [1]. En bloc resection was defined as the resection of the tumor in one piece without endoscopically visible residual tumor. R0 resection was defined as tumor resection with no histological evidence of cancer cells at the lateral and vertical margins. Curative resection was defined as en bloc resection with negative lateral and vertical margins and absence of LVI, when the lesion met one of the following criteria: (1) differentiated-type mucosal cancer of any size without ulceration; (2) differentiated-type mucosal cancer measuring ≤ 3 cm with ulceration; (3) differentiated-type cancer with minute submucosal invasion (invasion depth ≤ 500 µm) measuring ≤ 3 cm; or (4) undifferentiated-type mucosal cancer measuring ≤ 2 cm without ulceration. Non-curative factors were defined as any pathological or procedural findings that did not satisfy the above criteria for curative resection.

Histological types were classified into either the differentiated-type or the undifferentiated-type. The differentiated types included papillary adenocarcinoma and tubular adenocarcinoma while the undifferentiated type included poorly differentiated adenocarcinoma, poorly cohesive carcinoma and signet ring cell carcinoma [2]. Histological heterogeneity was defined when poorly differentiated adenocarcinoma or poorly cohesive carcinoma components coexisted as a minor portion of an ESD specimen (less than 50% of the tumor area), in accordance with Korean and Japanese guidelines [1,2,23].

Overall survival (OS) was defined as the period from the date of ESD to the date of death from any cause or the censoring date (8 May 2025). Data for OS were obtained using the national registry of medical insurance. Disease-free survival (DFS) was defined as the duration from the date of ESD to the date of detection of lymph node or distant recurrence and cancer-specific death. Metachronous recurrence was defined as cancer detected at a location other than the primary ESD site at least 12 months after ESD. Lesions detected within 12 months after ESD were defined as synchronous lesions.

### 2.4. Follow-Up After Treatment

Esophagogastroduodenoscopy (EGD) with biopsy was performed 2 months after ESD to confirm healing of the ESD-induced ulcer and to detect recurrence. Thereafter, EGD with biopsy and abdominal computed tomography were performed every 6 months for 3 years and annually for the following 2 years. The follow-up duration for recurrence was defined as the time from ESD to the last follow-up date of EGD or computed tomography.

### 2.5. Statistical Analysis

Categorical variables were analyzed using the chi-square test or Fisher’s exact test. Continuous variables were analyzed using Student’s *t*-test or Mann–Whitney test. Survival curves were plotted with the Kaplan–Meier method and the differences between the curves were tested using the log-rank test. Univariable logistic regression analyses were additionally performed to explore factors associated with lymph node metastasis (LNM). A *p*-value of <0.05 was considered statistically significant. All analyses were performed using SPSS version 29.0 (IBM Corp., Armonk, NY, USA).

## 3. Results

### 3.1. Clinicopathologic Characteristics of Mucosal EGC Patients with and Without Lymphatic Invasion

Among a total of 9117 EGC patients (9371 lesions) treated by ESD between 2001 and 2022, 7444 patients (7630 lesions) had EGC confined to the mucosa, of whom 154 patients (155 lesions) showed pathological evidence of LI. The incidence of LI in mucosal EGC after ESD was 2.1% (154/7444).

The clinicopathologic characteristics of mucosal EGC patients with and without LI are summarized in Table 1. Patients in the LI group were older (66.6 vs. 64.0 years, *p* < 0.001) and more frequently female (32.9% vs. 24.2%, *p* = 0.016). Tumors in the LI group were larger (2.0 cm vs. 1.5 cm, *p* < 0.001), with a higher proportion of lesions exceeding 2 cm (33.6% vs. 20.2%, *p* < 0.001). Within mucosal cancers, lesions with LI more frequently involved the muscularis mucosae rather than being confined to the lamina propria (93.5% vs. 52.6%, *p* < 0.001). Histological heterogeneity was also more common in the LI group (43.9% vs. 6.5%, *p* < 0.001).

### 3.2. Comparison of Characteristics in the Observation and Additional Surgery Group

Table 2 summarizes the characteristics of patients managed with observation and those who underwent additional surgery. The median follow-up duration in the observation and surgery groups was 53.5 months (interquartile range, 36–62 months) and 58.0 months (interquartile range, 46.5–59 months), respectively. Patients in the observation group were older (72.4 vs. 63.8 years, *p* < 0.001) and had a higher proportion of females compared with the surgery group (54.5% vs. 29.5%, *p* = 0.047). There were no significant differences between the two groups in tumor size, location, depth of invasion, histology, histological heterogeneity or ulcer status.

### 3.3. Long-Term Follow-Up Outcomes of Patients with Mucosal Early Gastric Cancer with Lymphatic Invasion as the Sole Non-Curative Factor

Figure 2 illustrates the follow-up outcomes of patients with mucosal EGC with LI as the sole non-curative factor. After excluding one patient who received radiotherapy, 117 patients were included in the outcome analysis; of these, 95 underwent additional surgery and 22 were managed with observation.

In the observation group, three patients showed metachronous recurrence (3/22, 13.6%), two showed LN enlargement during follow-up (2/22, 9.1%), and 17 had no recurrence (17/22, 77.3%) during a median follow-up of 53.5 months. Of the three patients with metachronous recurrence, two were curatively treated with ESD, whereas one underwent additional surgery, in whom LNM was confirmed in the surgical specimen. Among the two patients with LN enlargement on computed tomography, one underwent LN dissection, which revealed no metastasis, whereas the other could not undergo surgery because of poor general condition and was clinically diagnosed with LNM. Among the 95 patients who underwent surgery, LNM was identified in three patients (3.2%), and no recurrence was observed during a median follow-up of 58.0 months.

**Figure 3:**
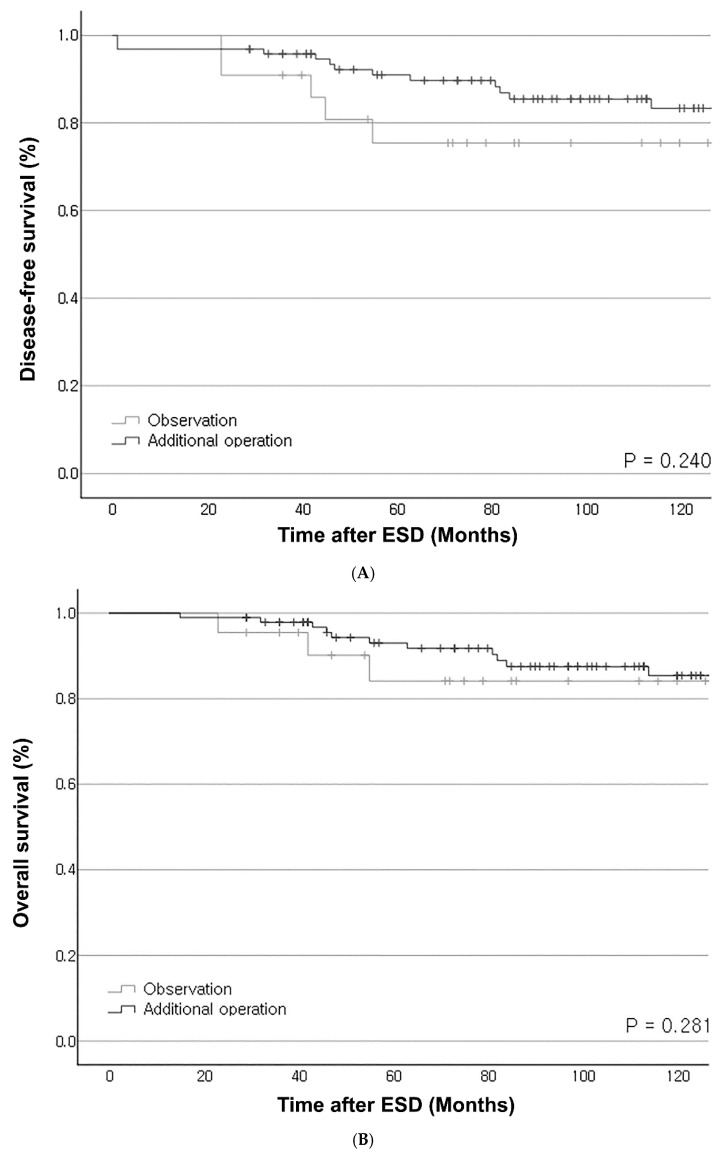
(**A**) Overall survival curves for patients in the observation group and the additional operation group. (**B**) Disease-free survival curves for patients in the observation group and the additional operation group.

Kaplan–Meier curves for OS and DFS showed no significant differences between the surgery and observation groups (*p* = 0.281 and *p* = 0.240, respectively) in Figure 3A,B.

### 3.4. Clinicopathologic Characterization and Curative-Criteria–Based Stratification of Lymph Node Metastasis in Patients with Lymphatic Invasion

The clinical characteristics of patients with pathologically confirmed or clinically suspected LNM are summarized in Table 3. Among the 117 patients, the overall incidence of LNM was 4.3% (5/117). LNM was pathologically confirmed in three patients (3.2%) in the additional surgery group, whereas two patients (9.0%) in the observation group were diagnosed with LNM either pathologically or clinically. One patient in the observation group was initially advised to undergo additional surgery but declined and subsequently underwent surgery for metachronous EGC detected 2 years after ESD, in which LNM was identified in the gastrectomy specimen. The histological features of the metastatic LN were identical to those of both the initial lesion and the metachronous EGC, and the primary origin of the metastasis could not be determined. Another patient in the observation group developed multiple enlarged lymph nodes on follow-up computed tomography 45 months after ESD; however, pathological confirmation was not feasible because of the patient’s deteriorated general condition, and the case was classified as clinically suspected nodal recurrence.

The distribution of pathological or clinically suspected LNM according to ESD curative resection criteria is summarized in Table 4. Clinical or pathological LNM was observed in 33.3% (1/3) of lesions confined to the lamina propria without ulceration and measuring > 2 cm, 1.4% (1/73) of lesions confined to the muscularis mucosae without ulceration and measuring ≤ 2 cm, and 9.1% (3/33) of lesions confined to the muscularis mucosae without ulceration and measuring > 2 cm. No pathological or clinical LNM was observed in differentiated lesions confined to the lamina propria measuring ≤ 2 cm without ulceration.

Exploratory logistic regression analysis was performed to identify factors associated with LNM in Table 5. In univariable analysis, old age and tumor size >2 cm were significantly associated with LNM.

## 4. Discussion

This retrospective study evaluated the long-term clinical outcomes of patients with mucosal EGC (pT1a) in whom LI was the sole non-curative factor after ESD. In this cohort, pathologically confirmed LNM or clinically suspected nodal recurrence was identified in 5 of 117 patients (4.3%), indicating that even in EGC confined to the mucosa, LI is associated with a clinically meaningful risk of LNM.

In the present study, the incidence of LI among mucosal EGC treated by ESD was 2.1% (154/7444). Lesions with LI were more frequently observed in older patients, females, tumors located in the antrum or angle, and lesions larger than 2 cm. Pathologically, LI was more commonly observed in lesions involving the muscularis mucosae rather than lamina propria, and histological heterogeneity was more frequent. These findings suggest that LI in mucosal EGC is not randomly distributed but is preferentially associated with specific clinicopathologic characteristics. Consistent with our findings, a recent Japanese study reported that LI in mucosal EGC was more common in older patients, tumors located in the lower stomach, lesions ≥30 mm in size, and mixed-type histology [14].

Although LI is generally considered to be strongly associated with LNM, data on the precise incidence of LNM in patients with mucosal EGC with LI as the sole non-curative factor are limited. Many previous studies have included cases with other non-curative factors or those with submucosal invasion. In surgical series that included such heterogeneous populations, markedly higher LNM rates have been reported, including a 48% (14/29) rate in Korea [25] and a 62.5% (5/8) rate in a Taiwanese cohort [26] in mucosal EGC with LI identified after gastrectomy. In contrast to these heterogeneous cohorts, a previous analysis of surgical data from our institution restricted to mucosal gastric cancer with LI as the sole non-curative factor reported no LNM in cases that met the absolute criteria for endoscopic resection (0%, 0/28) [13]. Moreover, because most patients with mucosal EGC are treated with ESD rather than surgery, surgical series may not fully represent the overall population of mucosal EGC and are therefore inherently subject to selection bias. In fact, post-ESD follow-up data from Japan demonstrated a substantially lower LNM rate of 2.5% (1/40) in pT1a patients with LI [14]. Given these discrepancies, our study is particularly meaningful in that it represents the largest ESD-based cohort (*n* = 117) with LI as the sole non-curative factor and long-term follow-up. In the present cohort, the overall rate of pathologically confirmed or clinically suspected LNM was 4.3%. Even when limited to patients who underwent immediate additional gastrectomy after ESD, pathologically confirmed LNM was identified in 3 of 95 patients (3.1%), indicating a clinically meaningful risk. These findings contrast with the view that observation alone may be sufficient for mucosal EGC when LI is the sole non-curative factor. Although the eCura system classifies lymphatic invasion as a high-risk non-curative factor requiring additional surgery, evidence specifically focused on mucosal EGC without other accompanying non-curative factors has been limited. Our findings provide long-term outcome data in this highly selected subgroup and support the clinical relevance of LI even in mucosa-confined EGC. It should be acknowledged that surgical treatment itself is associated with potential morbidity and mortality. However, perioperative mortality following gastrectomy for gastric cancer in Korea has been reported to be 1% [27]. Therefore, except in patients of very advanced age or those with severe comorbidities, additional surgical treatment may be reasonably considered for mucosal EGC with LI.

In the previously mentioned Japanese study [14], no LNM was observed in the pure differentiated-type mucosal EGCs without histological heterogeneity after ESD (*n* = 25). In contrast, in our cohort, pathologically confirmed LNM was identified after additional gastrectomy even in two patients without histological heterogeneity. This finding suggests that factors other than histological heterogeneity may contribute to the risk of LNM in mucosal EGC with LI. Accordingly, we further evaluated LNM risk according to curative resection criteria, including tumor size and depth of mucosal invasion. When stratified by these factors, no pathological or clinical LNM was observed in lesions measuring ≤ 2 cm and confined to the lamina propria. These findings suggest that tumor size and the depth of mucosal invasion—particularly involvement of the muscularis mucosae—should be carefully considered when assessing the risk of LNM. They may also help refine risk stratification within current guideline recommendations for non-curative ESD, particularly in selected low-risk patients with LI as the sole non-curative factor.

Emerging evidence suggests that tumor microenvironmental and microbial factors may also influence gastric carcinogenesis and metastatic potential [28,29]. Although the present study focused on established histopathological parameters, incorporation of these emerging biological factors may further improve future risk stratification in mucosal EGC.

The strength of this study is the relatively long median follow-up of 58.0 months and a large single-center cohort focusing exclusively on mucosal EGC with LI as the sole non-curative factor. Nevertheless, several limitations should be acknowledged. This was a retrospective, single-center study with a relatively small observation group (*n* = 22), which may limit generalizability and statistical power. In addition, the small number of metastatic events limited robust subgroup analyses and the ability to identify independent predictors of LNM through multivariable analysis. Decisions regarding additional surgery versus observation were not randomized and were influenced by patient age, comorbidity, and clinical judgment, potentially introducing selection bias. Therefore, comparisons of long-term outcomes between the surgery and observation groups should be interpreted cautiously. The diagnosis of LI may have been subject to interobserver variability, as immunohistochemical staining was selectively performed when LI was equivocal, which may have introduced variability or underestimation in LI assessment. In addition, clinically suspected metastatic recurrence was not always pathologically confirmed, raising the possibility of misclassification. Furthermore, in patients with metachronous EGC, attribution of metastatic recurrence to the index lesion may have been uncertain. Finally, although the follow-up duration was substantial, longer-term follow-up may be required to fully capture late recurrences; most EGCs are shown to recur within five years after curative surgery [30,31]. However, additional cases of recurrence could potentially develop over a more extended period.

## 5. Conclusions

LI in mucosal EGC is associated with a clinically meaningful risk of LNM. However, in carefully selected patients—particularly those with differentiated lesions measuring ≤ 2 cm and confined to the lamina propria—careful surveillance may be considered within an individualized clinical context, taking into account patient comorbidity and surgical risk.

## Figures and Tables

**Table 1:** Comparison of clinicopathological characteristics between mucosal EGC patients with and without lymphatic invasion.

	Lymphatic Invasion (+) (*n* = 155)	Lymphatic Invasion (−) (*n* = 7475)	Total (*n* = 7630)	*p*-Value
Age (mean ± SD)	66.6 ± 8.7	64.0 ± 10.1	64.0 ± 10.0	<0.001
Sex				0.016
Female	51 (32.9%)	1810 (24.2%)	1861 (24.4%)	
Male	104 (67.1%)	5665 (75.8%)	5769 (75.6%)	
Size (mean ± SD)	2.0 ± 1.1	1.5 ± 1.0	1.5 ± 1.0	<0.001
≤2 cm	103 (66.5%)	5962 (79.8%)	6065 (79.5%)	
>2 cm, ≤3 cm	28 (18.1%)	995 (13.3%)	1023 (13.4%)	
>3 cm	24 (15.5%)	518 (6.9%)	542 (7.1%)	
Location				<0.001
Antrum/angle	137 (88.4%)	5349 (71.6%)	5486 (71.9%)	
High-body/fundus/cardia	5 (3.2%)	583 (7.8%)	588 (7.7%)	
Low-body/Mid-body	13 (8.4%)	1501 (20.1%)	1514 (19.8%)	
Remnant stomach	0 (0.0%)	42 (0.6%)	42 (0.6%)	
Depth				<0.001
Lamina propria	10 (6.5%)	3540 (47.4%)	3550 (46.5%)	
Muscularis mucosa	145 (93.5%)	3932 (52.6%)	4077 (53.4%)	
Mucosa *	0 (0.0%)	3 (0.0%)	3 (0.0%)	
Histology				1.000
Differentiated	149 (96.1%)	7174 (96.0%)	7323 (96.0%)	
Undifferentiated	6 (3.9%)	301 (4.0%)	307 (4.0%)	
Histology heterogeneity				<0.001
No	87 (56.1%)	6990 (93.5%)	7077 (92.8%)	
Yes	68 (43.9%)	485 (6.5%)	553 (7.2%)	
Ulcer				0.083
No	153 (98.7%)	7457 (99.8%)	7610 (99.7%)	
Yes	2 (1.3%)	18 (0.2%)	20 (0.3%)	
Margin				0.580
NA	0 (0.0%)	37 (0.5%)	37 (0.5%)	
Negative	148 (95.5%)	7166 (95.9%)	7314 (95.9%)	
Positive	7 (4.5%)	272 (3.6%)	279 (3.7%)	
Vascular Invasion				<0.001
Negative	153 (98.7%)	7473 (100.0%)	7626 (99.9%)	
Positive	2 (1.3%)	2 (0.0%)	4 (0.1%)	
En bloc				0.843
En bloc resection	152 (98.1%)	7287 (97.5%)	7439 (97.5%)	
Piecemeal resection	3 (1.9%)	188 (2.5%)	191 (2.5%)	
Bleeding				0.328
No	153 (98.7%)	7254 (97.0%)	7407 (97.1%)	
Yes	2 (1.3%)	221 (3.0%)	223 (2.9%)	
Perforation				1.000
No	153 (98.7%)	7372 (98.6%)	7525 (98.6%)	
Yes	2 (1.3%)	103 (1.4%)	105 (1.4%)	

* Cases in which the depth of invasion could not be subclassified due to unavailable specimens. NA, not assessable (margin status could not be determined due to piecemeal resection); SD, standard deviation.

**Table 2:** Comparison of clinicopathologic characteristics between patients followed up with and without additional operation.

	Observation (*n* = 22)	Operation (*n* = 95)	Total (*n* = 117)	*p*-Value
Age	72.4 ± 7.3	63.8 ± 8.0	65.4 ± 8.6	<0.001
Sex				
Female	12 (54.5%)	28 (29.5%)	40 (34.2%)	0.047
Male	10 (45.5%)	67 (70.5%)	77 (65.8%)	
Size	1.7 ± 0.8	2.0 ± 1.1	1.9 ± 1.0	0.179
>2 cm	6 (27.3%)	30 (31.6%)	36 (30.8%)	0.890
≤2 cm	16 (72.7%)	65 (68.4%)	81 (69.2%)	
Location				
Antrum/angle	20 (90.9%)	85 (89.5%)	105 (89.7%)	0.788
High-body/Cardia	0 (0%)	2 (2.1%)	2 (1.7%)	
Low-body/Mid-body	2 (9.1%)	8 (8.4%)	10 (8.5%)	
Depth				
Lamina propria	3 (13.6%)	5 (5.3%)	8 (6.8%)	0.351
Muscularis mucosa	19 (86.4%)	90 (94.7%)	109 (93.2%)	
Histology				
Differentiated	21 (95.5%)	93 (97.9%)	114 (97.4%)	1.000
Undifferentiated	1 (4.5%)	2 (2.1%)	3 (2.6%)	
Histology heterogeneity				
No	16 (72.7%)	54 (56.8%)	70 (59.8%)	0.259
Yes	6 (27.3%)	41 (43.2%)	47 (40.2%)	
Ulcer				
No	22 (100.0%)	93 (97.9%)	115 (98.3%)	1.000
Yes	0 (0.0%)	2 (2.1%)	2 (1.7%)	
Follow-up duration (months)	53.5 (36–62)	58.0 (47–59)	58.0 (44–59)	0.170

Note: Values are presented as number (%), mean ± standard deviation, or median (interquartile range).

**Table 3:** Clinicopathologic characteristics of patients with pathologically confirmed lymph node metastasis after additional surgery and those with clinically suspected lymph node recurrence during follow-up.

No.	Age	Sex	Endoscopic Finding		Pathological Finding					Surgical Finding		Specific Consideration
			Location	Shape	Size (cm)	Histology	HH	Depth	Ulcer	Surgery Type	LNM	
1	67	M	LB LC	IIa + IIb	3.6	MD	No	LP	No	STG-II	1/34	
2	80	F	Angle PW	IIa + IIc	3.2	MD	No	MM	No	STG-II	1/25	
3	65	M	LB AW/GC	IIc	2.6	MD	Yes	MM	No	STG-I	1/29	
4	86	F	Antrum AW	I	3.0	Papillary	No	MM	No	STG-I	1/24	Initially advised to undergo surgery, but the patient declined. A metachronous EGC was detected 2 years later, and surgery was subsequently performed.
5	80	M	Antrum GC	IIa	1.6	MD	No	MM	No			Multiple enlarged lymph nodes were noted on follow-up gastric CT 45 months after ESD, but pathologic confirmation was not obtained due to the patient’s deteriorated condition.

No, number; HH, histological heterogeneity; LNM, lymph node metastasis; LB, low-body; LC, lesser curvature; PW, posterior wall; AW, anterior wall; GC, greater curvature; MD, moderately differentiated; Papillary, papillary adenocarcinoma; LP, lamina propria; MM, muscularis mucosa; STG-II, subtotal gastrectomy Billroth II; STG-I, subtotal gastrectomy Billroth I.

**Table 4:** Distribution of pathologic and clinically suspected nodal recurrences according to ESD curative-criteria subgroups in patients with lymphatic invasion as the sole non-curative factor.

Depth	Ulceration	Differentiated Type		Undifferentiated Type	
LP	(−)	≤2 cm	>2 cm		
		0/5	1/3		
MM	(−)	≤2 cm	>2 cm	≤2 cm	>2 cm
		1/73	3/33	0/3	0
	(+)	≤3 cm	>3 cm		
		0/2	0		

LP, lamina propria; MM, muscularis mucosa. Subgroup analyses were based on small sample sizes and should be interpreted cautiously.

**Table 5:** Univariable logistic regression analysis of risk factors associated with lymph node metastasis.

	Univariable Analysis		
	Odds Ratio	95% CI	*p*-Value
Age	1.197	0.041–1.377	0.012
Sex (male vs. female)	0.770	0.123–4.809	0.780
Tumor size (>2 cm vs. ≤2 cm)	10.000	1.076–92.936	0.043
Depth (MM vs. LP)	0.267	0.026–2.716	0.264
Histologic heterogeneity (present vs. absent)	0.359	0.039–3.314	0.366

CI, confidence interval; LP, lamina propria; MM, muscularis mucosa. Results should be interpreted cautiously because of the small number of metastatic events.

## Data Availability

The datasets used and/or analyzed during the current study are available from the corresponding author on reasonable request and with approval from the Institutional Review Board due to privacy and ethical restrictions.

## Abbreviations

EGC: Early gastric cancer; ESD: Endoscopic submucosal dissection; LNM: Lymph node metastasis; LN: Lymph node; LVI: Lymphovascular invasion; LI: Lymphatic invasion; RT: Radiotherapy; OS: Overall survival; DFS: Disease-free survival; EGD; Esophagogastroduodenoscopy.
